# Enhancing pharmacists' engagement and collaboration in sport and exercise medicine: An intervention mapping study using the behaviour change wheel

**DOI:** 10.1016/j.rcsop.2025.100619

**Published:** 2025-05-31

**Authors:** Alison D. Hooper, Jodie Marquez, Beata Bajorek, Joyce M. Cooper, David Newby

**Affiliations:** a School of Biomedical Sciences & Pharmacy, College of Health, Medicine & Wellbeing, University of Newcastle, Callaghan, NSW, Australia; b School of Health Sciences, College of Health, Medicine & Wellbeing, University of Newcastle, Callaghan, NSW, Australia; c Callaghan, NSW, Australia. College of Medicine & Dentistry, James Cook University, Cairns, QLD, Australia

**Keywords:** Sports pharmacy, Sport and exercise medicine, Pharmacists, Interprofessional collaboration, Physiotherapy, Behaviour change wheel, BCW, Behaviour change techniques, COM-B model, Behaviour change

## Abstract

**Background:**

Pharmacists are accessible, trusted healthcare professionals who are well-positioned to contribute to Sport and Exercise Medicine (SEM), yet their roles remain underutilised. Previous research using the Capability, Opportunity, Motivation-Behaviour (COM—B) model identified behavioural barriers and enablers influencing pharmacists' engagement in SEM and collaboration with physiotherapists.

**Objective:**

To design a theory-informed intervention toolkit to enhance pharmacists' engagement in SEM and support pharmacist–physiotherapist collaboration, using the Behaviour Change Wheel (BCW) framework.

**Methods:**

A structured, three-stage intervention mapping approach guided by the BCW was used. This included: (i) understanding behaviour and identifying influencing factors using COM-B analysis; (ii) identifying appropriate intervention functions and supporting policy categories; and (iii) selecting Behaviour Change Techniques (BCTs) and preferred modes of delivery based on the APEASE criteria.

**Results:**

Pharmacists' engagement in SEM is influenced by multiple barriers, including unclear role definitions, limited training and systemic constraints such as time and remuneration. Key enablers include pharmacists' medicines expertise, accessibility and motivation to collaborate. Six intervention functions and five policy categories were identified as suitable. Fifteen BCTs (e.g., goal setting, social support, prompts/cues) were selected to inform intervention content and delivery strategies, with various modes of delivery proposed.

**Conclusion:**

The BCW framework provided a structured method for developing an intervention toolkit aimed at enhancing pharmacists' engagement in SEM and collaboration with physiotherapists. The resulting strategies address key behavioural determinants and offer a foundation for future implementation. However, as the study focused on intervention design rather than implementation, further research is needed to assess the feasibility and real-world impact of the proposed strategies.

## 1 Background

### 1.1 Pharmacists' scope in sport and exercise medicine

Sport and Exercise Medicine (SEM) focuses on promoting physical activity and addressing related health concerns across all age groups, including recreational participants, elite athletes and those who may benefit from increased physical activity.1., 2., 3., 4 As recognition of the health benefits of physical activity has grown, so too has the need for multidisciplinary collaboration in SEM.^5^ Pharmacists, as trusted and highly accessible healthcare professionals, are well-positioned to contribute to SEM through provision of advice about medicines, non-pharmacological management and collaborative care.6., 7, 8 While the National Competency Standards Framework for Pharmacists (2016) acknowledges that sport and exercise-related care falls within pharmacists' scope of practice, their roles in SEM have not been clearly defined. Existing literature and training programs primarily focus on specialised areas of “sports pharmacy,” such as anti-doping in elite sports, while broader contributions in community and multidisciplinary contexts remain largely underexplored.9., 10, 11, 12 One recent qualitative study by Hooper et al. used the Capability, Opportunity, Motivation and Behaviour (COM—B) framework to explore pharmacists' engagement in SEM, including collaboration with physiotherapists.^8^ However, no Australian studies to date have examined how pharmacists' perceptions of their roles, scope of practice, or behavioural factors influencing their engagement in SEM could inform the design of interventions to support and enhance their involvement in multidisciplinary SEM care.

Given these gaps, aligning pharmacists' emerging roles in SEM with physiotherapist-led care pathways presents a timely and necessary opportunity for collaborative practice.

### 1.2 Physiotherapists and medication guidance

Physiotherapists, on the other hand, hold a well-established and widely recognised role in SEM and are often the initial point of contact for consumers seeking care.^2^^,^^13^ Beyond their expertise in injury management, evidence suggests that physiotherapists may at times be called upon to offer guidance on the use of over-the-counter medications and supplements.^7^^,^14., 15, 16 This may present challenges, particularly when physiotherapists work in settings with limited support from medical or pharmacist colleagues.^16^^,^^17^ The Australian Physiotherapy Association has advocated for autonomous prescribing rights for registered physiotherapists, with a focus on collaboration with pharmacists to ensure the safe and effective management of medications.^18^ Despite this, there is limited research exploring collaborative practice between pharmacists and physiotherapists within SEM contexts in Australia, particularly in the delivery of holistic patient care.

### 1.3 Behavioural determinants and theoretical frameworks

The first phase of this research, guided by the COM-B model (Capability, Opportunity, Motivation – Behaviour), conceptualises pharmacists' engagement in SEM as a behaviour shaped by their capability, opportunity, and motivation.^8^ By examining these components, the study aimed to uncover the behavioural factors influencing Australian pharmacists' engagement in SEM, including their collaboration with physiotherapists. Semi-structured interviews revealed key barriers and enablers, offering valuable insights for enhancing pharmacists' involvement in SEM.

The COM-B analysis revealed that pharmacists confidently identified as medicines experts in SEM but faced knowledge gaps in non-pharmacological strategies, sports supplements, and anti-doping practices, largely due to limited training opportunities. While frequently responding to SEM-related queries from diverse consumer groups, pharmacists often felt under-resourced, particularly in community settings where they commonly served as a triage point. Barriers such as limited resources, unclear role boundaries, time constraints and inadequate remuneration limited their ability to engage in their full scope of SEM-related care. Despite these challenges, pharmacists showed enthusiasm for expanding their role in SEM, particularly through enhanced collaboration with physiotherapists and support for autonomous physiotherapist prescribing.^8^

Building on these findings, the second phase of this research applies the Behaviour Change Wheel (BCW) framework to design a tailored intervention toolkit aimed at enhancing pharmacists' engagement in SEM, including collaboration with physiotherapists. The BCW offers a systematic approach for identifying effective intervention strategies by linking behavioural determinants to specific intervention functions and policy categories.^19^, ^20^, 21. In this phase, the COM-B model continues to underpin the intervention development process, with a focus on translating insights from the first phase into practical strategies for change.

This phase specifically explores how evidence-based Behaviour Change Techniques (BCTs)^19^, ^20^, 21. can be integrated into targeted interventions to address barriers and strengthen facilitators identified in the initial research.

The aim of this study was to design an evidence-based intervention toolkit to enhance pharmacists' engagement in SEM, including collaboration with physiotherapists, by applying the BCW framework. Three specific objectives aligned with the BCW stages were:

1. To draw on the results of a parallel research stream to understand pharmacist behaviour in SEM contexts by identifying capability, opportunity and motivation factors (COM-B analysis)
2. To identify relevant intervention functions, policy categories, and Behaviour Change Techniques (BCTs) to target the identified behavioural factors.
3. To develop a roadmap of intervention strategies and preferred delivery methods for future implementation and evaluation to optimise pharmacist participation in SEM, support pharmacist-physiotherapist collaboration and improve access to care and outcomes for consumers within the SEM landscape

## 2 Methods

### 2.1 Study design

The aim of this intervention mapping study was to apply the Behaviour Change Wheel (BCW) framework to develop an intervention toolkit designed to enhance pharmacists' engagement in SEM, including their collaboration with physiotherapists. The study followed the eight-step BCW process, structured across three stages:

**Stage 1: Understand the behaviour:** Define the problem in behavioural terms, identify and specify the target behaviour(s), and determine what needs to change using COM-B analysis.

**Stage 2:** Identify intervention options: Map the behavioural diagnosis to relevant intervention functions and policy categories using the APEASE criteria.

**Stage 3:** Identify content and implementation options: Select Behaviour Change Techniques (BCTs) and preferred modes of delivery for the proposed intervention toolkit.

These objectives ensured continuity with previous research while translating behavioural insights into practical strategies for change. Earlier phases of this broader research program included a systematic literature review and a qualitative study that applied the COM-B model to explore pharmacists' engagement in SEM. Semi-structured interviews with Australian pharmacists revealed key behavioural barriers—including limited SEM-specific training, unclear role boundaries, and lack of formal referral pathways—as well as enablers such as strong medicines expertise, high accessibility, and motivation to expand their role. These findings formed the behavioural diagnosis that underpins this study and justify the application of the BCW framework to develop a targeted intervention.^7^^,^^8^

#### 2.1.1 Theoretical framework

**Fig. 1:**
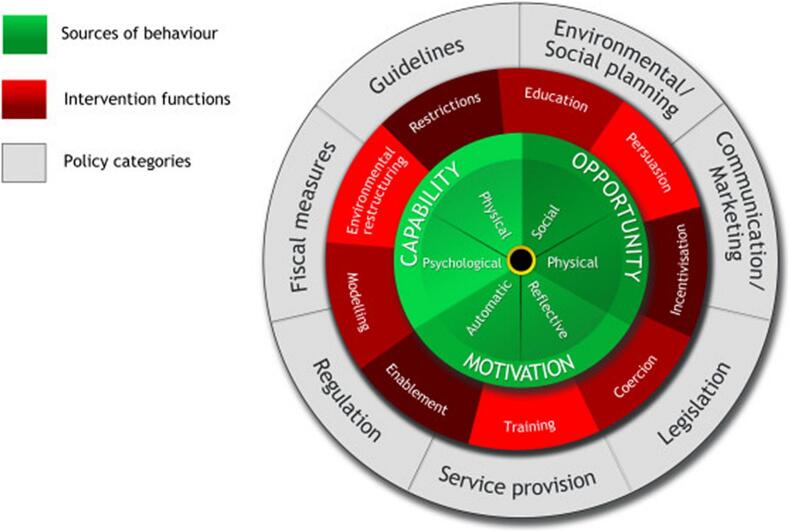
The Behaviour Change Wheel. Michie S, Atkins L, West R. (2014) The Behaviour Change Wheel: A Guide to Designing Interventions. London: Silverback Publishing. www.behaviourchangewheel.com.^20^

**Fig. 2:**
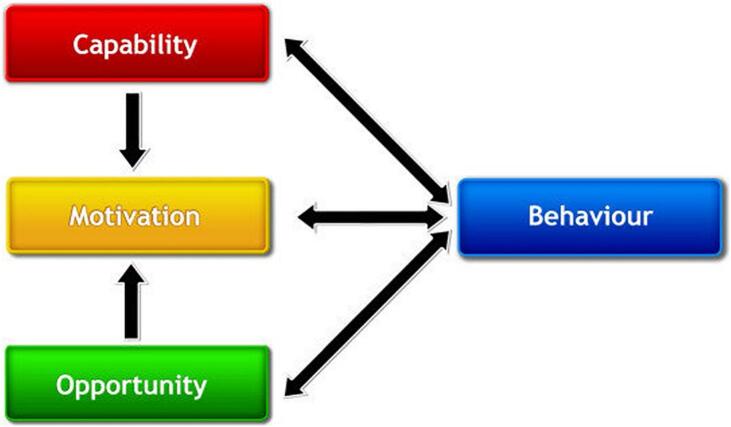
The COM-B model of behaviour. Michie S, Atkins L, West R. (2014) The Behaviour Change Wheel: A Guide to Designing Interventions. London: Silverback Publishing. www.behaviourchangewheel.com.^20^

The BCW (Fig. 1), which outlines an eight-step process for the design of interventions, formed the theoretical underpinning for this study.^19^, ^20^, 21. The BCW has, at its core, the COM-B model (Fig. 2), a behavioural framework used to explore and understand behaviour. The COM-B model highlights that behaviour is central to a complex, interconnected system shaped by an individual's or group's capability (physical and/or psychological), opportunity (social and/or physical) and motivation (reflective and/or automatic) to perform a specific behaviour.

Use of the BCW and it's constructs allowed us to draw on previous research employing the COM-B model to understand the behavioural factors influencing Australian pharmacists' capability, opportunity and motivation in engaging in sports and exercise medicine, including pharmacist-physiotherapist collaboration.^8^ The BCW was selected as the guiding framework due to its systematic and theory-informed approach to intervention design, which links behavioural diagnosis (via the COM-B model) to appropriate intervention functions and policy categories. This made it particularly well-suited to our research context, where pharmacists' engagement in SEM is shaped by complex individual, organisational, and professional influences. The BCW also offers practical advantages over frameworks that focus solely on evaluation or implementation, as it enables the structured development of targeted strategies to address identified behavioural barriers. Additionally, the COM-B components were woven throughout this study, framing the interpretation and contextualisation of findings. This integration ensured continuity between the prior research and the current phase, while supporting the design of a theoretically grounded and contextually relevant intervention toolkit for pharmacists in SEM.^19^^,^^20^

**Fig. 3:**
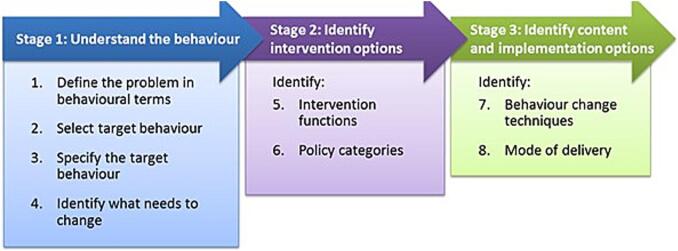
Steps in the Behaviour Change Wheel intervention design process. Michie S, Atkins L, West R. (2014) The Behaviour Change Wheel: A Guide to Designing Interventions. London: Silverback Publishing. www.behaviourchangewheel.com.^20^

Each step of the BCW was systematically applied following the structure recommended by Michie et al. (Fig. 3).^20^ Each component of behaviour change aligns with nine intervention functions: education, training, persuasion, enablement, incentivisation, restrictions, coercion, modelling and environmental restructuring; and seven policy strategies, including environmental/social planning, communication/marketing, regulation, legislation, guidelines, service provision and fiscal measures.^20^

Behaviour Change Techniques (BCTs), which represent the active components of interventions, were identified to enable evaluation and replication in the design and assessment of interventions.^20^ After conducting a behavioural analysis and identifying intervention options, promising BCTs were selected to guide effective intervention design.

Habit formation is a necessary consideration when sustained behaviour change is the desired outcome. Further, developing automatic behaviours can reduce cognitive load, allowing time-poor pharmacists to consistently integrate SEM-related tasks into their workflow.^22^ While habit formation has proven effective in simpler tasks like hand hygiene, more complex behaviours such as engagement in SEM, including integration as part of the multidisciplinary SEM team, are less suited to automatic habit formation and require targeted strategies aimed at specific components of behaviour.^23^^,^^24^ Incorporating a range of BCTs that target various intervention functions help to reinforce desired behavioural patterns and encourage these tasks to become part of standard practice.^22^

### 2.2 Research team

AH is a PhD candidate, holds a Master of Pharmacy and a Bachelor of Physiotherapy degree, has 15 years' experience as a pharmacist in Australia and 2 years' experience as a registered physiotherapist. DN, JC and BB are experienced pharmacy practice researchers and registered pharmacists. JM is a registered physiotherapist and experienced researcher.

BB is experienced in applying behaviour change theory, and DN, JC, BB and JM are experienced in qualitative methodology.

DN, JC and BB provided expertise and input from a pharmacy perspective, and JM from a physiotherapy perspective.

### 2.3 Stage 1: understand the behaviour

#### 2.3.1 Step 1: defining the problem in behavioural terms

The initial step involved conceptualising pharmacist engagement in SEM, including pharmacist-physiotherapist collaboration, as a behaviour, and defining the problem in behavioural terms.^20^ The research team conducted a systematic review of the literature exploring current and potential roles for pharmacists in SEM.^7^ This, along with findings from the first phase of this research involving qualitative interviews of Australian pharmacists and COM-B analysis,^8^ informed the researchers' views of barriers and enablers to pharmacists' engagement. The research team consisting of both pharmacists and physiotherapists, convened to agree on the answers to the following questions:

1. What is the behaviour?
2. Where does the behaviour occur?
3. Who is involved in performing the behaviour?
4. What are common barriers to performing the behaviour?

#### 2.3.2 Step 2: select the target behaviour(s)

Behaviours do not exist in isolation; they occur within the context of other behaviours, both of the same individual and others, forming part of a broader system.^20^ Therefore, following the definition of the problem, the next step involved systematically identifying a wide range of behaviours that could enhance pharmacists' engagement in SEM. This initial list of behaviours was developed by drawing on:

1. A systematic review of the literature to understand barriers, enablers, and current gaps in pharmacist engagement in SEM.^7^
2. Findings from the semi-structured interviews with 14 Australian pharmacists, which explored their perceptions and experiences within SEM, including pharmacist-physiotherapist collaboration.^8^
3. Personal experience and expertise within the research team, including perspectives from both pharmacy and physiotherapy disciplines.

Possible target behaviours were considered by the research team to reach agreement. The second task involved deciding which behaviours to target, considering the following four factors as recommended by the BCW^20^^,^^21^:

1. **Impact**: how much of an impact changing the behaviour will have on desired outcome?
2. **Feasibility**: how likely it is that the behaviour can be changed (when considering likelihood of change being achieved, think about the capability, opportunity and motivation to change of those performing the behaviour)?
3. **Interdependence**: how likely it is that the behaviour (or group of behaviours) will have a positive or negative impact on other, related behaviours?
4. **Measurability**: how easy it will be to measure the behaviour?

Based on these criteria, behaviours were rated as:

1. Very promising as a target behaviour.
2. Quite promising as a target behaviour.
3. Unpromising but worth considering as a target behaviour.
4. Not acceptable as the target behaviour.

#### 2.3.3 Step 3: specify the target behaviour

Once the target behaviours are selected, they are then specified in greater detail, considering context in which the behaviour occurs, including who needs to perform the behaviour, what the persons need to do differently, and when, where, how, and with whom they will do it. If we were unclear about the nature of pharmacists' engagement in sport and exercise medicine, we considered varying scenarios of practice to identify key characteristics. This approach helped to clarify distinct aspects of pharmacists' engagement and practice in SEM and pinpoint specific areas for behaviour change, including opportunities for collaboration with physiotherapists.

#### 2.3.4 Step 4: identifying what needs to change

This step focused on understanding what needs to change. In an earlier phase of the research, we conducted a detailed analysis of semi-structured interviews, informed by a systematic review of the literature and researcher clinical and research experience, and mapped themes to components of the COM-B model to identify factors required for behaviour change.^8^ This research complemented Step 4 by employing qualitative methods to explore pharmacists' and physiotherapists' perspectives on sports pharmacy, including pharmacist-physiotherapist interprofessional collaboration, across urban and rural/remote settings. Interviews with practicing pharmacists were viewed as necessary in grounding future interventions in real-world experiences and practice environments, ensuring their relevance and applicability. The results of this study pertaining to Step 4 are outlined in the Results section of this paper.

### 2.4 Stage 2: identify intervention options

#### 2.4.1 Step 5: identify intervention functions

In this step, relevant intervention functions were identified by systematically mapping them to the COM-B components, following the BCW framework. Each potential intervention function was evaluated using the APEASE criteria: Affordability, Practicability, Effectiveness and cost-effectiveness, Acceptability, Side effects and safety and Equity.^20^ This structured approach ensured that selected intervention functions were feasible, impactful and aligned with the study objectives.

#### 2.4.2 Step 6: identify policy categories

Seven policy categories are defined in the BCW framework^20^:

• Communication/marketing: using print, electronic, telephonic or broadcast media
• Guidelines: creating documents that recommend or mandate practice. This includes all changes to service provision
• Fiscal measures: using the tax system to reduce or increase the financial cost
• Regulation: establishing rules or principles of behaviour or practice
• Legislation: making or changing laws
• Environmental/social planning: designing and/or controlling the physical or social environment
• Service provision: delivering a service

Policy categories that could deliver intervention functions according to the BCW framework were mapped to intervention functions and assessed against the APEASE criteria to determine their suitability.

### 2.5 Stage 3: identify content and implementation options

#### 2.5.1 Step 7: identify behaviur change techniques

The final stage in the BCW framework is to identify the most appropriate behaviour change techniques (BCTs) that could result in the desired behaviour change. BCTs serve as the active components of interventions and provide a structured foundation for evaluating their effectiveness. The BCW highlights the BCTs most commonly associated with each intervention function, drawing on the Behaviour Change Technique Taxonomy version 1 (BCTT_V1_): a comprehensive framework of 93 BCTs developed through international expert consensus.^20^^,^^25^ We compiled a list of the most frequently used BCTs for the selected intervention functions and determined the suitability of each using the APEASE criteria. The selected BCTs were then mapped to intervention functions and COM-B components.

#### 2.5.2 Step 8: identify mode of delivery

The final step of the BCW involves developing a delivery framework informed by a recognised taxonomy of delivery modes.^20^ In the context of our study, we also drew on literature addressing interventions aimed at changing healthcare professional behaviour to guide the development of the framework.^26^ Each delivery mode category was evaluated using the APEASE criteria, with decisions finalised through consensus among the research team.

## 3 Results

This section presents the findings from the application of the BCW framework, building upon previously identified behavioural barriers and enablers from our earlier COM-B analysis.^8^ The results are presented according to the three BCW stages: (1) understanding the target behaviour, (2) identifying relevant intervention options, and (3) selecting content and delivery mechanisms. Each stage contributes directly to the development of a comprehensive intervention toolkit to support pharmacists' engagement in SEM, including collaboration with physiotherapists.

### 3.1 Stage 1: understand the behaviour

#### 3.1.1 Step 1: define the problem in behavioural terms

The responses agreed upon for the questions outlined in Step 1 are summarised below.

*What is the behaviour?*

Pharmacists engagement in, or provision of healthcare services within, the multidisciplinary field of sport and exercise medicine (SEM).

*Where does the behaviour occur?*

Various healthcare and sporting settings including, but not limited to: community pharmacies, hospital emergency departments, inpatient settings including preparation for discharge, and care provided in sporting contexts ranging from local sporting clubs to international, multi-sport competitions such as the Olympic Games.

*Who is involved in performing the behaviour?*

Ahpra-registered pharmacists practicing in an Australian healthcare or sporting setting.

*What are common barriers to performing the behaviour?*

Key barriers identified in the literature and through semi-structured interviews are described in Table 1. Barriers were described by pharmacists practicing across various practice settings in Australia^8^ and around the world.^7^

**Table 1:** Summary of barriers to pharmacists' engagement in SEM.

Barrier	Description
**Limited formalisation and recognition of pharmacists' roles in SEM, despite a broad scope of frequent SEM-related queries and consumer expectations.**	
Broad scope of SEM-related queries	The broad scope of SEM-related queries was identified as both an enabler and a barrier to pharmacists' engagement in SEM. Although their accessibility, frequent interactions and consumer expectations create opportunities, pharmacists' capability may be constrained by a lack of confidence in their knowledge, which may not fully align with the diverse range of services demanded.^8^
Consumer expectations	A key barrier is the gap between consumer expectations for evidence-based advice on SEM-related products and pharmacists' perceived limited knowledge and confidence to meet these expectations, particularly for products stocked in community pharmacies. Consumer expectations can also be considered an enabler contributing to opportunity.^8^
Lack of clearly defined scope for pharmacists' practice in SEM	While roles within SEM align with the National Competency Standards Framework for Pharmacists (2016),^9^ pharmacists have expressed concerns about overstepping professional boundaries. This ambiguity creates uncertainty about their role in providing SEM-related advice and services, hindering their confidence and ability to engage fully in this area.^8^
Limited description of roles for pharmacists in SEM in literature	The literature describing pharmacists' roles in ‘sports pharmacy’ predominantly emphasises doping prevention and control for competitive or elite athletes, often neglecting their broader and more routine responsibilities within everyday practice.^7^
**Insufficient training and educational opportunities to support pharmacists providing SEM-related services**	
Limited inclusion of SEM reported in undergraduate training	Pharmacists identified the limited emphasis on SEM in undergraduate education as a key barrier, leaving them feeling unprepared to address the types of presentations they encounter in practice.^8^
Lack of knowledge about evidence-based non-pharmacological strategies	A recurring theme in the literature and pharmacist interviews highlights pharmacists' uncertainty and lack of confidence in addressing non-pharmacological sports-related queries, such as strapping techniques, joint support, and equipment recommendations, due to insufficient guidance and knowledge. Additionally, the literature suggests that pharmacists often lack awareness and expertise in doping and anti-doping practices. Those without specific training or qualifications frequently struggle to accurately classify substances as prohibited or permitted in sports.^7^^,^^8^
Limited opportunities for professional development reflecting broad scope of pharmacists' roles in SEM	Limited opportunities for professional development are available to pharmacists in the context of SEM, particularly in addressing the broad scope of their roles. Despite an interest in acquiring SEM-specific skills, pharmacists report insufficient access to tailored training programs, leaving gaps in their preparedness to provide comprehensive SEM-related services.^8^
Lack of evidence to support recommendations concerning use of supplements	A lack of evidence supporting the efficacy and safety of supplements was identified in the literature as a significant barrier to pharmacists' ability to confidently provide recommendations.^7^
**Limited opportunities for interdisciplinary collaboration, including pharmacist-physiotherapist collaboration**	
Infrequent inclusion of pharmacists in multi-disciplinary SEM teams	Pharmacists have highlighted limited opportunities for inclusion in multidisciplinary SEM teams, identifying this as both a significant barrier to collaboration and an untapped opportunity to enhance patient care through integrated, team-based approaches.^8^
Lack of formalised referral pathways	Pharmacists frequently refer patients to physiotherapists and other health professionals, such as physiotherapists and podiatrists; however, these referrals are typically informal. The lack of structured referral processes has been identified by pharmacists as a significant barrier to effective interprofessional collaboration in SEM.^8^
Perceived lack of understanding and appreciation by other healthcare professionals regarding pharmacists' potenital contributions to SEM.	A lack of awareness and understanding of pharmacists' expertise among other healthcare professionals was identified as a barrier to pharmacists' practice and effective collaboration in SEM. Pharmacists reported in semi-structured interviews that while other professionals were not opposed to pharmacists' involvement, they often lacked insight into the value pharmacists could bring to medical decisions. This gap in understanding, coupled with concerns about the appropriateness of medication requests following physiotherapy consultations, highlights the need for improved communication and greater pharmacist input in multidisciplinary care.^8^
**Systemic barriers**	
Time and workload burden in community pharmacy	The perception of ‘lack of time’, particularly in relation to concerns about high workload, was identified as a barrier to pharmacists' engagement in SEM both in the literature^7^ and semi-structured interviews with pharmacists,^8^ particularly in relation to pharmacists practicing in a community setting. Lack of time was also identified as a barrier to counselling on sports supplements and physical activity promotion by pharmacists^27^^,^^28^ and to pharmacists adopting their full scope of practice.^29^ Addressing the challenges of time constraints aligns with recommendations from the National Institute for Health and Care in the UK, which advocate for tailoring behaviour change interventions in health care to different levels of intensity, including “very brief,” “brief,” “extended brief,” and “high intensity” approaches.^30^
Need for appropriate remuneration	Pharmacists raised concerns about their compensation, underscoring the importance of adequate remuneration for time spent in consumer consultations, particularly as these interactions may not necessarily result in product sales in community pharmacy. This was seen as especially critical when providing detailed SEM advice, which was seen to involve time-intensive consultations and highly personalised care.^8^

#### 3.1.2 Step 2: select the target behaviour(s)

The selected behaviours focus on enhancing pharmacists' proactive engagement in SEM while addressing systemic barriers and promoting interdisciplinary collaboration.

##### 3.1.2.1 Target behaviour

Pharmacists engage in provision of a broad scope of SEM services, including collaboration with physiotherapists as part of the multidisciplinary SEM team.

##### 3.1.2.2 Specific sub-behaviours

The target behaviour is considered to incorporate the following sub-behaviours in order to address identified barriers:

- Providing SEM-related consultations (e.g., advising on medicines, injury prevention).
- Engaging in SEM-specific training and education to address gaps in knowledge and build confidence and skills in providing evidence-based SEM services, including non-pharmacological strategies and product/device recommendations.
- Establishing and utilising structured referral pathways with physiotherapists and other SEM stakeholders while engaging in multidisciplinary initiatives to foster collaboration and knowledge-sharing.
- Pharmacists empowered to overcome systemic barriers, such as managing time constraints and advocating for remuneration, to allocate sufficient time and resources for SEM-related services in community pharmacies.

By retaining a broad scope, this approach ensures that interventions can address various facets of pharmacists' roles in SEM while aligning with the selected criteria for prioritisation. These behaviours were identified as *very promising*, as they align with pharmacists' existing scope of practice, address key barriers and have the potential to incorporate identified enablers.

#### 3.1.3 Step 3: specify the target behaviour

Specifying the target behaviours involved a multifaceted approach aimed at enhancing the level of engagement of Australian pharmacists in SEM, with a specific focus on collaborative efforts with physiotherapists. This process is outlined in Table 2.

**Table 2:** Specifying the target behaviour.

Target behaviour	Pharmacists engaging in the provision of a broad scope of SEM services, including collaboration with physiotherapists as part of a multidisciplinary SEM team.
*Who* needs to perform the behaviour?	Pharmacists across various settings, including community pharmacies, hospitals and professional sporting environments.
*What* do they need to do differently to achieve the desired change?	Pharmacists need to expand their engagement in SEM by providing evidence-based consultations, participating in multidisciplinary collaborations, addressing systemic barriers such as time constraints and remuneration, and pursuing SEM-specific training to build confidence and skills.
*When* do they need to do it?	During routine practice, whenever SEM-related opportunities or consumer queries arise, and through regular professional development activities.
*Where* do they need to do it?	In their respective practice settings, such as pharmacies, hospitals or other healthcare or sporting environments, and in collaborative spaces, whether in-person or virtual, for multidisciplinary teamwork and education.
How *often* do they need to do it?	Consistently, as part of their daily practice and professional growth, ensuring sustained engagement with SEM services and collaboration efforts.
With *whom* do they need to do it?	With consumers, physiotherapists, and other SEM stakeholders in multidisciplinary teams, as well as with professional and sporting organisations and policymakers to address systemic barriers.

### 3.2 Step 4: identify what needs to change

Barriers and enablers were identified to all six elements of the COM-B model. The results of the behavioural diagnosis are presented in Table 3, with examples drawn from the parallel semi-structured interview research stream.

**Table 3:** Behavioural diagnosis using themes from semi-structured interviews.

COM-B Component	Behavioural target	Barrier or Enabler
Capability (Physical)	Pharmacists have the skills to provide SEM-specific services and care (e.g. compounding, first aid, measuring for braces and mobility aids, vaccinations).	Enabler
	Despite receiving consumer queries for SEM-related services such as strapping, pharmacists lack the training or skills to provide these services which may also fall outside of their scope of practice.	Barrier
	Pharmacists possess a skillset that could be better utilised within the multidisciplinary SEM context.	Enabler
Capability (Psychological)	Pharmacists have the knowledge and expertise to provide medicines advice to consumers of SEM (e.g. recommendations and advice regarding medicines to consumers and prescribers, drug safety, recommending medicines for a broad range of minor ailments, travel medicine, doping prevention and control).	Enabler
	Pharmacists are confident in their ability to provide medicines-related advice in SEM.	Enabler
	Some pharmacists may be familiar with anti-doping resources while others are uncertain about where to access reliable information on drugs in sports in a timely manner.	Both
	Pharmacists' extensive knowledge and understanding of polypharmacy and its impact on physical capacity may be an underutilised resource within SEM.	Enabler
	Pharmacists possess the knowledge and understanding necessary to assess needs and direct individuals to appropriate care in a timely manner.	Enabler
	Pharmacists possess the knowledge and expertise to address potentially inappropriate medication requests from consumers following physiotherapy consultations and have the potential to offer more proactive input, ensuring recommendations are safe, effective and tailored to individual patient needs.	Enabler
	Pharmacists occasionally share their expertise on medicines in SEM by facilitating interdisciplinary training sessions for other healthcare professionals.	Enabler
	Pharmacists expressed a desire for hands-on training in practical skills like strapping and brace fitting.	Enabler
	Pharmacists lack knowledge and confidence in providing advice on non-pharmacological SEM-related queries, such as strapping techniques, joint support and equipment recommendations.	Barrier
	Pharmacists lack resources to support their responding to non-pharmacological SEM-related queries.	Barrier
	Pharmacists have the knowledge and expertise to support physiotherapists prescribing.	Enabler
	Pharmacists' have the knowledge and expertise to contribute meaningfully to the multidisciplinary SEM team.	Enabler
Opportunity (Physical)	Consumers regularly visit pharmacies seeking advice and support for SEM-related queries.	Enabler
	Pharmacists receive a broad scope of SEM-related queries.	Enabler
	Pharmacists engage with a diverse demographic of consumers across the lifespan, providing opportunities to address varied SEM-related needs.	Enabler
	Pharmacists' high accessibility supports their role in SEM by making their services readily available to consumers.	Enabler
	Pharmacists are often relied upon to triage SEM-related presentations, assessing needs and directing individuals to appropriate care.	Enabler
	Pharmacists lack the physical resources needed to facilitate a structured referral to physiotherapists and other healthcare professionals.	Barrier
	Certain practice environments, such as hospital settings and professional sporting contexts where pharmacists have established roles within multidisciplinary SEM teams, are more conducive to interdisciplinary collaboration. In contrast, co-location of community pharmacies with other practitioners can sometimes, but not consistently, enhance opportunities for collaboration.	Enabler
	Limited professional development opportunities reflecting broad scope of pharmacists' roles in SEM.	Barrier
	Pharmacists recognise the value of interprofessional education in SEM, particularly when conducted alongside physiotherapists and other healthcare professionals, to enhance collaboration and shared understanding.	Enabler
	Pharmacists may prefer the flexibility and accessibility of online education and training options in SEM.	Enabler
	Pharmacists in rural or remote locations may view face-to-face training as a barrier due to limited accessibility and logistical challenges.	Barrier
	Pharmacists describe time constraints as a barrier to delivering SEM-related services, particularly in a community pharmacy setting.	Barrier
	Limited resources to support pharmacists providing non-pharmacological advice and recommendations in SEM.	Barrier
	No established remuneration model to compensate community pharmacists for the time spent providing SEM consultations.	Barrier
Opportunity (Social)	Consumers perceive pharmacists as a trusted source of SEM-related healthcare and advice, addressing a wide range of concerns.	Enabler
	Pharmacists have the opportunity to provide SEM-related services and support through partnerships with local sporting clubs.	Enabler
	Consumers expect pharmacists to provide evidence-based advice on SEM-related products and services, particularly those stocked in the pharmacy.	Both
	Pharmacists frequently recommend that consumers consult a physiotherapist.	Enabler
	Pharmacists rely on informal referral pathways to connect consumers with physiotherapists and other health professionals, such as podiatrists.	Barrier
	There is a lack of effective collaboration between physiotherapists and pharmacists, which can lead to medication recommendations that may not fully align with individual patient needs.	Barrier
	Other healthcare professionals may have limited awareness of pharmacists' expertise and their potential to contribute positively to SEM.	Barrier
	Opportunities for pharmacists within multidisciplinary SEM teams are limited.	Barrier
	Opportunity for pharmacists to collaborate with physiotherapists to support and enhance autonomous physiotherapist prescribing.	Enabler
	Pharmacists recognise a need for their involvement in multidisciplinary teams to enhance care and outcomes.	Enabler
Motivation (Reflective)	Pharmacists' high accessibility sometimes leads to a sense of obligation to provide advice that may extend beyond their scope of practice or professional boundaries, creating potential challenges in maintaining role clarity.	Barrier
	Pharmacists are committed to delivering evidence-based advice in SEM, even when faced with limited access to reliable evidence-based resources.	Both
	Pharmacists are enthusiastic about interdisciplinary collaboration in SEM.	Enabler
	Pharmacists have a strong interest in pursuing professional development opportunities across the full scope of their potential roles in SEM to enhance their knowledge and skills.	Enabler
	Pharmacists seek training and education opportunities in SEM that address the broad scope of queries they encounter, expressing concern that existing modules are overly focused on anti-doping.	Both
	Pharmacists express a desire for inter-professional learning in SEM.	Enabler
	Pharmacists may experience uncertainty about the limits of their role in SEM, expressing concerns about overstepping professional boundaries or practicing outside their scope in areas such as mouthguards, strapping, and injury recovery.	Barrier
	There is a lack of clear guidelines to assist pharmacists in identifying when referrals to other healthcare professionals are appropriate.	Barrier
	Pharmacists' motivation to engage in SEM consultations is hindered by the lack of remuneration for their time.	Barrier
	Pharmacists are enthusiastic about playing a greater role in SEM in the future.	Enabler
	Pharmacists have a desire and ambition to be more involved in SEM settings.	Enabler
Motivation (Automatic)	Pharmacists are more likely to engage in SEM if they have a personal interest in sports (e.g., childhood history of participation in sports, regular involvement in physical activity or a passion for watching sports).	Both

### 3.3 Summary (stage 1)

This stage identified the behavioural targets and influencing factors shaping pharmacists' engagement in SEM. These insights directly informed the selection of intervention functions and guided the content development for the intervention toolkit.

### 3.4 Stage 2: identify intervention options

#### 3.4.1 Step 5: identify intervention functions

Six out of the nine BCW intervention functions met the APEASE criteria. Incentivisation, coercion and restriction did not meet the criteria in the context of pharmacists' engagement and integration into multidisciplinary SEM teams. The selected intervention functions were: Education, persuasion, training, environmental restructuring, modelling and enablement. The process of assessing and selecting intervention functions, and mapping these to COM-B components is shown in Table 4.

**Table 4:** Selecting intervention functions.

BCW intervention function	Definition^20^	Meets APEASE criteria?	Comments	COM-B component/s
Education	Increasing knowledge or understanding	Yes	Highly feasible and effective as first step to address knowledge gaps. Requires funding for materials, education sessions/module development, digital resources etc. Some pharmacists in rural or remote areas may face barriers to accessing face-to-face education opportunities.	Capability (Psych); Motivation (Refl)
Persuasion	Using communication to induce positive or negative feelings or stimulate action	Yes	Moderately useful to raise awareness and influence perceptions but requires support from other interventions. Persuasion needs to be carefully framed to avoid resistance or defensiveness. Can motivate stakeholders to integrate pharmacists but may not overcome systemic barriers without other interventions.	Motivation (Auto, Refl)
Incentivisation	Creating an expectation of reward	No	Not affordable or practical – affordability limits large-scale implementation. Implementing incentives also requires clear frameworks and funding.	*–*
Coercion	Creating an expectation of punishment or cost	No	Not suitable in this context. Unlikely to be accepted by pharmacists or SEM stakeholders. Potential to undermine professional relationships and could harm relationships and trust. Inaffordable and could disproportionately impact pharmacists with fewer resources or opportunities.	*–*
Training	Imparting skills	Yes	Highly effective for improving pharmacists' skills but requires funding. Could enhance pharmacists' capability and confidence to engage in SEM and participate in multidisciplinary SEM teams. Requires resources, coordination and access to training programs. Rural or regional pharmacists may face barriers to accessing hands-on training.	Capability (Phys, Psych); Opportunity (Phys); Motivation (Auto)
Restriction	Using rules to reduce the opportunity to engage in the target behaviour (or to increase the target behaviour by reducing the opportunity to engage in competing behaviours)	No	Not suitable in this context. High risk; may lead to unintended consequences e.g., restrict access to other types of health care. Creating and reinforcing restrictions would be complex and unethical.	*–*
Environmental restructuring	Changing the physical or social context	Yes	Highly effective for creating opportunities and fostering integration. Requires collaboration across healthcare settings to implement changes. May involve costs related to new roles or systems. Can apply to pharmacists' roles in SEM across all settings.	Opportunity (Phys, Soc); Motivation (Auto)
Modelling	Providing an example for people to aspire to or imitate	Yes	Useful for inspiring engagement but may work best when combined with other interventions. Pharmacists likely to respond positively to successful examples of integration. Case studies and success stories are easy and cost-effective to share.	Opportunity (Soc); Motivation (Auto)
Enablement	Increasing means/ reducing barriers to increase capability (beyond education and training) or opportunity (beyond environmental restructuring)	Yes	Highly effective for reducing barriers and improving integration but practicability depends on specific barriers targeted (e.g. time, funding/remuneration may require more complex considerations). May involve resource allocation or system-level changes. Targeted enablement strategies can address disparities (e.g. rural or remote settings).	Capability (Phys, Psych); Opportunity (Phys, Soc); Motivation (Auto)
Selected intervention functions:		Education, persuasion, training, environmental restructuring, modelling, enablement.		

These six intervention functions (education, persuasion, training, environmental restructuring, modelling, and enablement) were selected based on their alignment with the COM-B components identified in earlier phases and their feasibility as assessed using the APEASE criteria. Together, they form the functional backbone of the proposed intervention toolkit.

#### 3.4.2 Step 6: identifying policy categories

Of the seven BCW policy categories, five were selected to support the delivery of the intervention functions identified in Step 5. While fiscal measures are dependent on funding, they were included due to their potential usefulness, as the aim of this framework is to develop a comprehensive toolkit of possible interventions. Table 5 presents the assessment and selection of policy categories, along with examples of potential delivery mechanisms designed to enhance pharmacists' engagement in SEM, including collaboration with physiotherapists and other key stakeholders.

**Table 5:** Selection of policy categories.

Intervention function	COM-B component	Potentially useful policy categories	Meets APEASE criteria?	Potential delivery mechanisms
Education	Capability (Psych); Motivation (Refl)	Communication/ marketing	Yes	Develop SEM-specific online modules addressing pharmacists' skills gaps (e.g., evidence-based non-pharmacological advice for common SEM presentations); webinars, printable materials (consumer leaflets, posters) and indexed resources for timely access; disseminate evidence-based information and SEM publications through professional bodies, digital prompts and seminars; encourage pharmacists' participation in physical activity by sharing evidence-based health benefits.
		Guidelines	Yes	Develop and disseminate guidelines defining pharmacists' SEM roles, referral pathways and communication of red flags; guidelines to support autonomous physiotherapist prescribing with pharmacist collaboration; make guidelines accessible to pharmacists and SEM stakeholders through professional bodies; promote awareness of pharmacists' expertise in multidisciplinary SEM teams.
		Regulation	No; unlikely to be practical or acceptable in this context	–
		Legislation	No; not required for improving education in SEM	–
		Service provision	Yes	Provide structured guidance on using SEM-specific resources, including but not limited to anti-doping resources, during service delivery; establish pharmacist-specific resources to support their SEM services, tailored to non-pharmacological advice and recommendations.
Persuasion	Motivation (Auto, Refl)	Communication/marketing	Yes	Campaign materials; success stories; media outreach; targeted stakeholder meetings; communication from professional bodies; share evidence demonstrating pharmacists' value in SEM; include quotes from consumers and influential stakeholders to encourage pharmacist engagement and promote their expertise to other SEM professionals.
		Guidelines	Yes	Guidelines communicating pharmacists' value in SEM, including their integration into multidisciplinary teams and collaboration with physiotherapists to ensure holistic, safe and tailored care and advice.
		Regulation	No; not considered acceptable in this context	–
		Legislation	No; not necessary or practical	–
		Service provision	Yes	Pilot programs integrating pharmacists into SEM teams to demonstrate value; develop resources to prompt consumers to seek SEM advice from pharmacists (e.g., posters, leaflets); establish initiatives to educate consumers on pharmacists' SEM capabilities.
Training	Capability (Phys, Psych); Opportunity (Phys); Motivation (Auto)	Guidelines	Yes	Develop explicit guidelines for pharmacists on recommending non-pharmacological products relevant to their scope of practice, such as key elements of strapping, brace fitting and mobility aid measurement and usage; ensure guidelines are supported by the inclusion of SEM-specific training for pharmacists in professional standards and practice frameworks.
		Fiscal measures	Moderate; dependent on funding	Subsidies for pharmacist participation in SEM training; funding for inter-professional training initiatives.
		Regulation	No; unnecessary for training uptake	–
		Legislation	No; unlikely to drive implementation	–
		Service provision	Yes	Develop accessible training programs tailored to pharmacists' SEM-specific needs, offered through flexible face-to-face and online formats (training should include non-pharmacological products commonly stocked in pharmacies, within pharmacists' scope of practice, such as strapping techniques, brace fitting and mobility aid measurement and use); incorporate interprofessional training with physiotherapists to enhance collaboration, focusing on non-pharmacological advice, injury management, and practical skills; promote pharmacists as trusted sources of medicines-related information and expertise for other SEM professionals.
Environmental restructuring	Opportunity (Phys, Soc); Motivation (Auto)	Guidelines	Yes	Include protocols for pharmacist integration into SEM teams; guidelines for referral pathways and interdisciplinary collaboration in SEM; include pharmacists in future guidelines supporting autonomous physiotherapist prescribing.
		Fiscal measures	Moderate; dependent on funding	Provide financial support for SEM team restructuring, referral pathway establishment and infrastructure.
		Regulation	No; not practicable as reliant on external bodies	–
		Legislation	No; not considered practical, acceptable or necessary in this context	–
		Environmental/social planning	Yes	Establish SEM clinics or create collaborative spaces for pharmacists and physiotherapists; develop infrastructure to support referral pathways and improve inter-professional collaboration; establish regional networks for sharing SEM resources and referrals.
Modelling	Opportunity (Soc); Motivation (Auto)	Communication/marketing	Yes	Share case studies, success stories, and role models to inspire integration; establish ambassador programs showcasing successful pharmacist involvement in SEM; share pharmacists' experiences about positive effects of regular physical activity.
		Service provision	Yes	Embed pharmacists in pilot SEM projects or professional sports teams; establish mentorship programs and online peer support networks to foster collaboration; role-modelling opportunities through structured collaboration with experienced SEM practitioners.
Enablement	Capability (Phys, Psych); Opportunity (Phys, Soc); Motivation (Auto)	Guidelines	Yes	Develop guidelines addressing barriers to pharmacist engagement in SEM; create resources integrated with IT systems for seamless use during SEM service delivery.
		Fiscal measures	Moderate; dependent on funding	Provide funding for pharmacists' integration into SEM, such as subsidies for inter-professional collaboration and training.
		Regulation	No; not practicable as reliant on external bodies	–
		Legislation	No; not considered practical, acceptable or necessary in this context	–
		Environmental/social planning	Yes	Create workflows and physical or digital spaces to enable pharmacist inclusion in SEM; ensure resource usability across settings; establish professional networks and communities of practice to share resources and peer support; encourage participation in physical activity for both social and health benefits.
		Service provision	Yes	Establish pharmacist-led SEM clinics in sports or community settings; provide workplace-based programs to facilitate collaboration with SEM stakeholders; customise resources for various practice settings, ensuring accessibility and relevance.
Selected policy categories:	Communication/marketing, guidelines, service provision, environmental/social planning, fiscal measures			

### 3.5 Summary (stage 2)

The selected intervention functions and policy categories provide a framework for targeting the behavioural determinants identified in Stage 1. They establish the strategic direction of the intervention toolkit.

### 3.6 Stage 3: identify content and implementation options

#### 3.6.1 Step 7: identify behaviour change techniques

In order to identify which BCTs can deliver the identified intervention functions under the relevant policy categories, we identified 20 promising BCTs based on the most frequently used BCTs for each selected intervention function. These were then assessed according to the APEASE criteria in the context of this research, as shown in Table 6. Fifteen BCTs were selected. BCTTv1 No. 1.3 - Goal setting (outcome) - did not meet APEASE criteria as it was deemed impractical to obtain data representing outcome of pharmacists' engagement in real world settings and unlikely to be taken up by time-poor pharmacists concerned about workload,. Feedback on outcome(s) of behaviour (BCTTv1 No. 2.7) was considered impractical for similar reasons; further, measuring outcomes of pharmacists' engagement in SEM is likely to require follow up, including in simulated training scenarios. Self-minotoring of behaviour (BCTTv1 No. 2.3) was considered impractical and unlikely to be adopted by pharmacists due to identified workload concerns. Adding objects to the environment (BCTTv1 No. 12.5) in order to facilitate performance of the behaviour did not meet the APEASE criteria as it was considered unlikely to be effective in this context and dependent on funding (this BCT excludes prompts/cues and provision of information in a booklet or leaflet is insufficient according to the Taxonomy). Restructuring the physical environment (BCTTv1 No. 12.1) was considered impractical, unnecessary in this context and/or too costly.

**Table 6:** Assessment and identification of behaviour change techniques.

Intervention function	COM-B component	Selected policy categories	Potential Behaviour Change Techniques (BCTs)⁎	Meets APEASE criteria?	Examples of implementation of BCTs in the context of enhancing pharmacists' engagement in SEM including collaboration with physiotherapists
Education	Capability (Psych); Motivation (Refl)	Communication/ marketing Guidelines Service provision	5.3 Information about social and environmental consequences	Yes	Explain that provision of SEM-related services leads to enhanced access to care for consumers. Explain that enhanced collaboration pathways with physiotherapists enhances continuity of care for consumers and encourages opportunities for inter-professional learning Inform that actively pursuing SEM-related professional development opportunities enhances the ability to practice at the full scope within SEM. Explain the potential social benefits of regular and sufficient physical activity.
			5.1 Information about health consequences	Yes	Explain how the misuse of over-the-counter medications can lead to adverse health outcomes, such as delayed injury recovery, adverse effects and drug-drug interactions, emphasising the pharmacist's role in optimising medicines use in SEM. Explain how optimising the use of non-pharmacological over-the-counter products, such as selecting the most appropriate strapping tape or ensuring the correct adjustment and measuring of mobility aids, can promote faster recovery and prevent further injury. Explain that communication channels between pharmacists and physiotherapists create opportunities for pharmacists' input in SEM, such as helping prevent inappropriate use of over-the-counter medicines and offering guidance on how concurrent medications may influence therapy outcomes, such as the mechanism of injury and recovery. Explain the health benefits of regular and sufficient physical activity.
			2.2 Feedback on behaviour	Yes	Access to a pharmacist mentor with expertise in SEM to observe and/or provide personalised feedback on how a pharmacist educates patients about the proper selection of non-pharmacological products like strapping tape or mobility aids. Conducting role-play where pharmacists educate a simulated SEM consumer, followed by facilitator feedback. Recommend use of pedometer or smart-tracking technology to encourage and reinforce engagement in physical activity.
			2.7 Feedback on outcome(s) of the behaviour	No; impractical to measure outcomes of pharmacists' engagement in SEM, including in simulated scenarios. Requires follow-up. Unlikely to be accepted by time-poor pharmacists concerned about workload.	
			7.1 Prompts/cues	Yes	Place a sticker/sign near SEM-related medicines and products such as pain relief gels and strapping tape with messages like: *“Ask your pharmacist for advice on proper use and injury management.”* List of local physiotherapist practices and contact details. Implement screen savers or pop-up reminders on pharmacy point-of-sale software that prompt staff to offer pharmacists' input when scanning relevant products. Use of a checklist for common SEM-related consultations. Provide pharmacists with pocket-sized cue cards summarising key counselling points for SEM-related consultations, such as guidance on selecting the most appropriate strapping tape for different injuries and the importance of mobility aid adjustments. Display posters or cue cards in counselling rooms with key SEM education points, serving as a reminder for pharmacists during patient consultations. Post visual reminders in staff areas, such as “Remember to educate patients about SEM product selection and use”. SMS reminders to reinforce points such as key SEM-related counselling points or the benefits of physical activity.
			2.3 Self-monitoring of behaviour	No; impractical and unlikely to be adopted by pharmacists due to identified workload concerns	
Persuasion	Motivation (Auto, Refl)	Communication/ marketing Guidelines Service provision	9.1 Credible source	Yes	Live or recorded webinar/speech delivered by pharmacist/s with experience in SEM and/or physiotherapist working in SEM Consumer testemonies
			5.3 Information about social and environmental consequences	Yes	Share real-world case studies showing how pharmacists' input in SEM has positively influenced community health, such as improved injury recovery, return-to-sport or safer use of medicines. Introduce recognition programs showcasing pharmacists who have made a positive social impact through effective SEM engagement, such as partnerships with local sporting teams, reinforcing the value of this behaviour.
			5.1 Information about health consequences	Yes	Share stories of patients who experienced negative consequences, such as delayed recovery or worsened condition due to improper use of SEM-related products, emphasising the importance of pharmacist intervention. Develop leaflets or pocket-sized cards summarising key health consequences of incorrect SEM medicine or product use, serving as a quick reference during consultations.
			2.2 Feedback on behaviour	Yes	Create a visible board in the pharmacy where pharmacists receive public recognition for their contributions to SEM, using positive feedback to motivate continued involvement. Create consumer feedback cards to collect feedback specifically about consumers' interactions with pharmacists regarding SEM products and services.
			2.7 Feedback on outcome(s) of the behaviour	No; impractical to measure outcomes of pharmacists' engagement in SEM	
Training	Capability (Phys, Psych); Opportunity (Phys); Motivation (Auto)	Guidelines Fiscal measures Service provision	6.1 Demonstration of the behaviour	Yes	Conduct hands-on training workshops where experienced pharmacists or physiotherapists demonstrate effective SEM-related counselling, such as selecting the right strapping tape or measuring/adjusting mobility aids. Develop a series of instructional videos showcasing best practices for SEM counselling, including how to identify patient needs and offer guidance effectively. Record and share role-playing scenarios where a pharmacist models a successful SEM-related consultation, which may incorporate triage/referral/collaboration with a physiotherapist or providing input as part of the multidisciplinary SEM team. Develop interactive e-learning modules where pharmacists watch demonstration videos and then practice skills in virtual patient interactions.
			4.1 Instruction on how to perform the behaviour	Yes	Create structured quick-reference guidelines and/or ‘decision trees’ for pharmacists to follow when responding to specific SEM-related queries, incorporating guidance on management and ‘red flags’ and prompts to facilitate triage and referral. Design e-learning modules with guided instructions, quizzes, and virtual patient interactions to reinforce key concepts in responding to SEM-related queries. Develop case studies with guided instructions and sample responses, asking pharmacists to work through a SEM-related scenario.
			2.2 Feedback on behaviour	Yes	Use simulated patient interactions (face-to-face or virtual) where pharmacists respond to SEM-related queries and receive feedback from an pharmacist with experience in SEM or physiotherapist. Conduct follow-up meetings after training sessions where pharmacists can reflect on their progress and receive additional feedback based on their practical experience.
			2.7 Feedback on outcome(s) of behaviour	No; impractical to measure outcomes of pharmacists' engagement in SEM, including in simulated training scenarios	
			2.3 Self-monitoring of behaviour	No; impractical and unlikely to be adopted by pharmacists due to identified workload concerns	
			8.1 Behavioural practice/rehearsal	Yes	Organise structured role-playing exercises where pharmacists practice delivering SEM-related counselling to a simulated patient. Set up practice stations during workshops (face-to-face or virtual) where pharmacists rotate through different SEM-related scenarios, such as explaining the choice of strapping tape or fitting a mobility aid. Provide pharmacists with scripted SEM-related scenarios they can rehearse individually or in pairs.
Environmental restructuring	Opportunity (Phys, Soc); Motivation (Auto)	Guidelines Fiscal measures Environmental/ social planning	12.5 Adding objects to the environment	No; unlikely to be effective in this context and dependant on funding	
			7.1 Prompts/cues	Yes	Place a sticker/sign near SEM-related medicines and products such as pain relief gels and strapping tape with messages like: *“Ask your pharmacist for advice on proper use and injury management.”* List of local physiotherapist practices and contact details. Implement screen savers or pop-up reminders on pharmacy point-of-sale software that prompt staff to offer pharmacists' input when scanning relevant products. Provide pharmacists with pocket-sized cue cards summarising key counselling points for SEM-related consultations, such as guidance on selecting the most appropriate strapping tape for different injuries and the importance of mobility aid adjustments. Display posters or cue cards in counselling rooms with key SEM education points, serving as a reminder for pharmacists during patient consultations. Post visual reminders in staff areas, such as “Remember to educate patients about SEM product selection and use”. SMS reminders to reinforce key SEM-related counselling points.
			12.1 Restructuring the physical environment	No; impractical, unnecessary and/or costly in this context	
Modelling	Opportunity (Soc); Motivation (Auto)	Communication/ marketing Service provision	6.1 Demonstration of the behaviour	Yes	Invite physiotherapists or other SEM experts to deliver live demonstrations on effective patient education techniques for products like strapping tape or mobility aids. Share success stories from experienced pharmacists describing how they incorporate SEM education into their practice. Develop instructional videos where experienced pharmacists model counselling and/or inter-professional collaboration in SEM.
Enablement	Capability (Phys, Psych); Opportunity (Phys, Soc); Motivation (Auto)	Guidelines Fiscal measures Environmental/ social planning Service provision	3.1 Social support (unspecified)	Yes	Create pharmacist support groups or communities of practice where colleagues can share experiences and motivate each other to engage in SEM-related practice and professional development. Organise social events where pharmacists, physiotherapists and other SEM practitioners can build rapport and share professional experiences in a non-work setting.
			3.2 Social support (practical)	Yes	Provide toolkits, guidelines and/or quick-reference guides for effective SEM counselling. Encourage joint patient education sessions with pharmacists and physiotherapists to model optimal SEM practices, including collaboration. Facilitate pharmacists' direct access to SEM experts (e.g., physiotherapists) for practical advice on complex cases.
			1.1 Goal setting (behaviour)	Yes	Develop goals for the number of joint pharmacist-physiotherapist case discussions per quarter, focusing on coordinated patient care. Set goals for proportion of SEM encounters provided with a resource (e.g. leaflet and information about safe usage provided to 70 % of consumers purchasing mobility aids).
			1.3 Goal setting (outcome)	No; impractical to obtain data representing outcome of pharmacists' engagement in real world settings; unlikely to be taken up by time-poor pharmacists concerned about workload.	
			12.5 Adding objects to the environment	No; unlikely to be effective in this context and dependent on funding	
			1.2 Problem solving	Yes	Prompt pharmacists to consider barriers preventing them from engaging in SEM services and/or collaborating with physiotherapists and other healthcare professionals within SEM and consider ways in which these could be overcome. Establish communities of practice in SEM where pharmacists can share challenges faced during SEM counselling and discuss collective solutions. Provide self-reflection templates where pharmacists document challenging SEM counselling experiences and outline how they could approach the situation differently next time.
			1.4 Action planning	Yes	Encourage pharmacists to create individualised action plans for how they will improve engagement in SEM-related services in their practice, including when and how to initiate conversations with physiotherapists and other healthcare professionals. Encourage collaborative meetings between pharmacists and physiotherapists to co-develop action plans for shared patient care, such as role definitions referral processes. After SEM-related training sessions, have pharmacists outline how they will apply new skills in practice, including the resources they need and when they will start. Encourage pharmacists to create action plans addressing common barriers, such as limited SEM product knowledge, with steps for seeking support or additional training. Encourage pharmacists to organise and/or participate in local walking groups.
			2.3 Self-monitoring of behaviour	No; impractical and unlikely to be adopted by pharmacists due to identified workload concerns	
			12.1 Restructuring the physical environment	No; impractical and/or costly in this context	
			1.5 Review behaviour goal(s)	Yes	After SEM-related workshops, hold follow-up meetings where pharmacists review how effectively they've applied new strategies in their practice. Encourage pharmacists to maintain journals tracking their SEM-related behaviours and periodically review entries to identify trends and areas for improvement. Could be facilitated as part of a training program for pharmacists.
			1.7 Review outcome goal(s)	Yes	Encourage regular (e.g., annual) re-evaluation and refinement of outcome goals, allowing pharmacists to identify new or adjusted objectives tailored to their evolving practice and increased experience in SEM. Encourage regular multidisciplinary team reviews where pharmacists and physiotherapists collaboratively assess patient health outcomes, such as timely referrals and reductions in inappropriate medicine requests following physiotherapy consultations, to evaluate the impact of SEM practices and refine or develop new collaborative outcome goals.
Selected BCTs:	1.1, 1.2, 1.4, 1.5, 1.7, 2.2, 3.1, 3.2, 4.1, 5.1, 5.3, 6.1, 7.1, 8.1, 9.1				

⁎ Behaviour Change Techniques (BCTs) numbered according to the Behavioural Change Technique Taxonomy version 1 (BCTTv1) and ordered according to frequency of use for the given intervention function.^20^

Fifteen BCTs were identified and selected for inclusion in the intervention toolkit. These techniques were chosen for their strong theoretical linkage to the selected intervention functions and practical relevance to pharmacists' practice settings, as well as their feasibility and acceptability according to the APEASE criteria.

#### 3.6.2 Step 8: identify mode of delivery

Distance delivery was preferred as this ensured accessibility across both urban and rural/remote areas with flexibility to minimise disruption to workload. Internet-based approaches were preferred for widespread online resource delivery, accessible across urban and rural/remote areas. It offers flexibility around workload and other commitments and online resources can be developed to be compatible for desktops, laptops, tablets or mobile phones. However, the importance of face-to-face opportunities for practical skills training is acknowledged, with group settings favored for their practicality and ability to foster collaboration and peer support. Population-level delivery approaches were prioritised to maximise the accessibility of the intervention toolkit for Australian pharmacists, with digital strategies identified as the most suitable for broad reach and flexibility. Posters and leaflets were also considered viable for intervention delivery but may incur significant costs for printing and distribution. Digital resources present a promising alternative, contingent on stakeholder engagement for printing, display and active use. Articles and professional development activities in pharmacy-focused publications were also viewed as promising channels for dissemination. The BCW delivery framework is summarised in Table 7.

**Table 7:** Identification of modes of delivery for an intervention to enhance pharmacist engagement in SEM, including pharmacist-physiotherapist collaboration.

Mode of Delivery				APEASE Criteria Met?
Face-to-face	Individual			No; impractical, does not provide opportunity for collaboration and peer support
	Group			Moderate; dependant on funding availability for facilitators and venue. May be less accessible for rural and remote practitioners. However, it is optimal for delivering hands-on training for practical skills. Group settings promotes collaboration and peer support.
Distance	Population-level	Broadcast media	TV	No; not affordable nor applicable to majority of audience.
			Radio	No; not affordable nor applicable to majority of audience.
		Outdoor media	Billboard	Moderate; likely inaffordable and may be difficult to effectively target pharmacists. However, billboards or booths at conferences and professional events could be effective, depending on available funding.
			Poster	Yes; could be printed or digital. Printed posters not affordable, unless funding obtained. Effectiveness of digital posters relies heavily on uptake by stakeholders for printing, display and usage.
		Print media	Newspaper	Yes; while mainstream newspaper advertisements or articles are likely unaffordable and impractical, consumer-targeted articles could promote pharmacists' expertise in SEM and increase public awareness. Articles and professional development activities in pharmacy-specific professional publications may effectively reach a wide pharmacist audience but could involve publication fees. Potential for publication into physiotherapy-specific publications to enhance awareness and promote collaboration. This approach also offers the potential to incorporate multiple selected BCTs.
			Leaflet	Yes; could be paper or digital, aimed at consumers, pharmacists or physiotherapists. Printing and disseminating leafletd could be costly. Digital leaflets couls be easily distributed to a broad population but rely on stakeholder printing, display and usage of leaflets.
		Digital media	Internet	Yes; preferred platform for widespread delivery of online resource. Accessible to both urban and rural/remote pharmacists. Offers flexibility in delivery, accommodating workload demands and other commitments. Accessible via desktop/laptop (often used in workplaces), as well as tablet or mobile phone. Articles in professional publications also disseminated online (e.g. via email or member portal login).
			Mobile phone app	Moderate; development of a dedicated mobile phone application costly. Internet-based resources can be optimised for use on tablets and mobile phones, providing a more cost-effective and accessible alternative.
	Individual-level	Phone	Phone helpline	No; impractical without funding.
			Mobile phone text	Moderate; potentially costly. SMS messaging may be a useful tool for delivering prompts and cues.
		Individually accessed computer programme		No; expensive and impractical to develop purpose-built computer programme. Online resource significantly more accessible and does not rely on purchase/installation of computer program.
Selected mode/s of delivery	Preferred: Face-to-face (group), billboard, poster, newspaper (e.g. professional publication), leaflet, internet, mobile phone app, mobile phone text.			

### 3.7 Summary (stage 3)

The selection of BCTs and preferred delivery modes forms the practical foundation of the intervention toolkit, offering actionable strategies to support pharmacists' capability, opportunity, and motivation to engage in SEM and collaborate with physiotherapists.

## 4 Discussion

Pharmacists are well-positioned to play a significant role in SEM through their accessibility, expertise in medication management, and regular contact with consumers seeking SEM-related advice. They are uniquely placed to offer guidance on medicines, supplements, and over-the-counter products, triage and refer individuals appropriately, and contribute to multidisciplinary care alongside physiotherapists and other SEM professionals. However, barriers such as unclear role boundaries, limited training in SEM-specific skills, lack of formal referral pathways, and inadequate remuneration continue to hinder optimal engagement in this space.

This study applied the BCW framework as a roadmap to designing an intervention toolkit aimed at enhancing pharmacists' engagement in SEM and promoting collaboration with physiotherapists and the broader multidisciplinary SEM team. By building on findings from the COM-B analysis conducted in the first phase of this research,^8^ this study translated identified barriers and facilitators into practical strategies using evidence-based BCTs. Key insights from this phase highlight the importance of addressing capability, opportunity, and motivation factors to optimise pharmacists' involvement in SEM. Several barriers were identified. The perceived lack of formalisation and recognition of pharmacists' roles in SEM, despite a broad scope of frequent consumer queries and expectations, was noted as a key barrier. Limited education and training opportunities exist that reflect the broad scope of pharmacists' roles and presenting queries in SEM, particularly concerning non-pharmacological strategies. Additionally, limited opportunities for interdisciplinary collaboration and rare inclusion of pharmacists in multidisciplinary SEM teams, perceived lack of understanding and appreciation by other healthcare professionals regarding pharmacists' potential contributions to SEM, as well as lack of formalised referral pathways, further impact pharmacists' confidence and engagement. Systemic barriers such as time and workload concerns and limited remuneration were also identified.

Enablers identified included capability factors such as pharmacists' existing knowledge, expertise and unique skillset, particularly concerning the provision of medicines advice and managing related products. These capabilities could be further leveraged to support physiotherapists' prescribing in the future. Opportunity-related enablers included the frequent SEM-related queries received from a diverse consumer base and the high accessibility of pharmacists, positioning them well for deeper involvement and integration within multidisciplinary SEM teams. Motivation factors were also prominent, with pharmacists expressing a clear desire to upskill and deliver evidence-based SEM-related services, including a strong interest to expand their skills in non-pharmacological care areas, enhancing their contributions as integral members of the SEM care team.

To address barriers and build on existing enablers, intervention functions education, persuasion, training, environmental restructuring, modelling and enablement were selected. These strategies aim to create a supportive framework for pharmacists to build knowledge and skills to confidently engage in SEM practice while collaborating effectively with physiotherapists and the broader SEM team.

The systematic application of each step within the BCW framework, though extensive, was essential to ensure all components of effective behaviour change were thoroughly considered. Consistent with the BCW methodology, the process of selecting BCTs began with identifying those most frequently associated with the chosen intervention functions, followed by an assessment of their suitability using the APEASE criteria in the context of enhancing pharmacists' engagement in SEM, including pharmacist-physiotherapist collaboration.^19^, ^20^, 21. While the BCW approach supports prioritising the most frequently used BCTs for each intervention function, not reviewing the full set of 93 BCTs within the BCTTv1 taxonomy could risk overlooking techniques that might be both effective and novel for this area of practice.

It is well-established that passive approaches to behaviour change interventions are generally ineffective; education and training alone are unlikely to drive sustained changes in health practitioner behaviour.^31^ However, our research identified education and training as essential components within a broader, holistic intervention strategy to enhance pharmacists' engagement in SEM. By embedding BCTs targeting education and training within a multifaceted intervention, informed by behaviour theory and guided by the BCW framework, this approach is more likely to yield meaningful and sustained practice change compared to implementing a single intervention in isolation.^31^ BCTs that target education and training intervention functions as part of an intervention toolkit to enhance pharmacist engagement in SEM include information about health, social and environmental consequences, instruction on how to perform the behaviour, demonstration of the behaviour, prompts/cues, feedback on behaviour and encouraging self-monitoring of behaviour. Effective strategies include integrating educational resources into routine care, providing real-time decision support and utilising point-of-care prompts to encourage best practices. Professional development is further enhanced when strategies are supported by credible sources and designed to address identified barriers and facilitators of behavioural change. In addition to implementing these BCTs in the context of education and training, some are useful for addressing the remaining intervention functions identified in this study; selected BCTs include social support, goal setting, problem solving and action planning.^20^

The importance of social support and collaboration was evident throughout the findings. The inclusion of strategies such as peer mentoring, shared learning workshops and facilitation of interprofessional collaboration opportunities demonstrates the value of social and environmental restructuring in enhancing pharmacist engagement. Targeted approaches such as pharmacist-physiotherapist case reviews and co-delivered training sessions were considered practical and feasible strategies for supporting interdisciplinary collaboration.

The use of digital delivery was prioritised to ensure accessibility for both urban and rural/remote pharmacists, minimising workload disruptions while expanding the reach of the intervention toolkit. However, face-to-face opportunities were also acknowledged as critical for hands-on practical skills training, particularly for competencies such as mobility aid adjustments and strapping techniques. Printable resources such as posters and leaflets, as well as professional publications were also promising modes of delivery.

### 4.1 Limitations and future research

While the BCW framework provided a structured and theory-informed approach to intervention design, this study has several limitations. First, the findings reflect the perspectives of pharmacists only and do not capture the views of physiotherapists or other stakeholders in SEM teams. As a result, the desirability and feasibility of pharmacist-physiotherapist collaboration from the physiotherapists' perspective remains unknown. Second, while intervention strategies were mapped and prioritised using behavioural theory and the APEASE criteria, their feasibility and effectiveness have not yet been tested in real-world settings.

Future research should include pilot implementation of the proposed intervention toolkit to assess its impact on pharmacist engagement, interprofessional collaboration, and consumer health outcomes in SEM. Additionally, exploring the perspectives of physiotherapists and other SEM professionals will be essential to optimise interdisciplinary integration and ensure the acceptability and sustainability of collaborative models.

## 5 Conclusion

This study demonstrates how the BCW framework, underpinned by prior COM-B analysis, can be systematically applied to design a targeted intervention toolkit to enhance pharmacists' engagement in SEM, including collaboration with physiotherapists. Each stage of the process—understanding behaviour, selecting intervention functions and policy categories, and identifying behaviour change techniques—was grounded in previously identified behavioural determinants and directly informed the toolkit's structure and content.

By addressing individual, organisational, and systemic barriers while leveraging identified enablers, the intervention strategies presented offer a cohesive, theory-informed roadmap to support pharmacist engagement in SEM. This study provides a strong foundation for future implementation and evaluation of pharmacist-physiotherapist collaboration models in multidisciplinary SEM practice, with intervention strategies designed to enhance pharmacists' capability, opportunity, and motivation to engage meaningfully in SEM care.

## CRediT authorship contribution statement

**Alison D. Hooper:** Writing – review & editing, Writing – original draft, Visualization, Validation, Supervision, Software, Resources, Project administration, Methodology, Investigation, Formal analysis, Data curation, Conceptualization. **Jodie Marquez:** Writing – review & editing, Visualization, Validation, Supervision, Methodology. **Beata Bajorek:** Writing – review & editing, Visualization, Validation, Supervision, Methodology. **Joyce M. Cooper:** Writing – review & editing, Visualization, Validation, Supervision, Methodology. **David Newby:** Writing – review & editing, Visualization, Validation, Supervision, Project administration, Methodology.

## Ethics approval

Ethics approval to conduct this study was granted by the Human Research Ethics Committee at the University of Newcastle (Protocol number: H-2020-0075).

## Declaration of competing interest

Alison is an employee of the University of Newcastle. The research is funded under the Australian Government's Research Training Program (RTP). The remaining authors have nothing to declare.

## Glossary

Sport and Exercise Medicine (SEM): A multidisciplinary area of healthcare that aims to optimise sporting performance at all levels and promote physical activity as a tool for preventing and managing chronic conditions such as cardiovascular disease, diabetes, and arthritis. SEM supports individuals across all activity levels, from elite athletes to those increasing physical activity for better health outcomes.
Sports Pharmacy: A specialised area within pharmacy, largely focused on providing medication-related guidance to physically active individuals and athletes. This often includes advice on supplement use and adherence to anti-doping regulations.
COM-B Model: A behavioural framework that explains actions as the result of interactions between three elements: Capability (physical and psychological), Opportunity (social and physical), and Motivation (reflective and automatic), which interact to influence behaviour.
Behaviour Change Wheel (BCW): A theoretical framework that builds on the COM-B model, used to design interventions aimed at influencing health-related behaviours.
Behaviout Change Technique (BCT): a specific, evidence-based strategy used in behaviour change interventions. It is an active component that targets factors like knowledge, motivation, or environment to influence behaviour.
Behaviour Change Technique Taxonomy version 1 (BCTTv1): A comprehensive, standardised classification of 93 Behaviour Change Techniques, grouped into 16 categories. It provides a common language to identify, describe, and report the active components of behaviour change interventions.
APEASE Criteria: A set of principles used to assess the suitability of behaviour change interventions. The acronym stands for Affordability, Practicability, Effectiveness and cost-effectiveness, Acceptability, Side-effects/safety, and Equity. These criteria help determine whether an intervention is appropriate for real-world implementation.
Anti-Doping: A system of rules, procedures, and actions aimed at deterring and detecting the use of banned substances or methods in competitive sport.
Triage: A method of evaluating and ranking patients' health needs to determine the urgency of care, often including referrals to other health professionals—particularly in settings like community pharmacy.
Referral Pathways: Structured protocols that assist healthcare providers in appropriately directing patients to other services or professionals for further care or evaluation.
Non-Pharmacological Interventions: Health strategies that do not involve medication, such as physical activity, nutritional advice, use of braces or supports, and assistive devices; often provided by pharmacists within their practice scope.
